# The Legacy of Conventional Oil and Gas Development Outweighs Shale Gas Impacts on Stream Biodiversity

**DOI:** 10.1021/acsestwater.5c01413

**Published:** 2026-03-03

**Authors:** Ryan Olivier-Meehan, Ariel Levi Simons, Anirudh Prabhu, Elizabeth Carter, Ruta Basijokaite, Greg Lackey, Tao Wen

**Affiliations:** 1 Department of Earth and Environmental Sciences, 2029 Syracuse University, Syracuse, New York 13244, United States; 2 Institute of the Environment and Sustainability, 8783 University of California, Los Angeles, California 90095, United States; 3 Earth and Planets Laboratory, Carnegie Institution for Science, Washington, District of Columbia 20015, United States; 4 Department of Civil and Environmental Engineering, 2029 Syracuse University, Syracuse, New York 13244, United States; 5 Department of Civil, Environmental and Architectural Engineering, 1877 University of Colorado, Boulder, Colorado 80309, United States

**Keywords:** benthic macroinvertebrate, Appalachian Plateau, Marcellus Shale, network analysis, oil and gas development

## Abstract

Unconventional oil and gas development (UOGD) has expanded rapidly across the Appalachian Basin, raising concerns about its ecological effects. Although UOGD has been linked to water quality changes that may affect stream biota, a generalized relationship with stream biota remains unresolved. We evaluated how UOGD and conventional oil and gas development (COGD) influence benthic macroinvertebrate (BMI) communities across the Marcellus Shale region of Pennsylvania using one of the most comprehensive statewide BMI datasets, integrating delineated catchments, oil–gas records, and more than 6800 BMI samples. Linear mixed-effect models and co-occurrence network analyses were used to quantify effects on BMI taxonomy, functionality, and network structure while controlling for confounding factors. We found no significant association between UOGD intensity and BMI diversity metrics, whereas COGD intensity was correlated with reduced richness, diversity, and biotic integrity—comparable to the impact of developed land cover. Network analyses indicated altered community structures near both development types: COGD was linked to larger, more connected networks dominated by pollution-tolerant taxa, while UOGD was associated with larger but more fragmented networks. Both forms of development were tied to increases in generalist taxa and declines in specialists. Overall, UOGD exerted limited but detectable ecological effects, whereas COGD imposed broader stress on stream communities.

## Graphical abstract

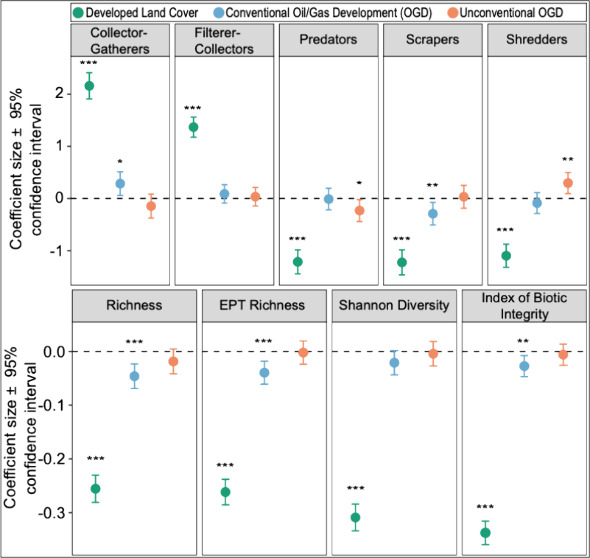

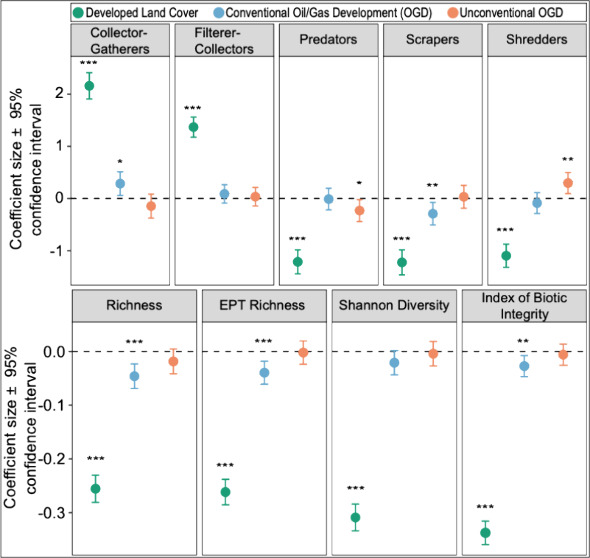

## Introduction

Energy development has expanded rapidly in the United States of America (USA), transforming landscapes and raising questions about its ecological costs. In particular, the advent of unconventional oil and gas development (UOGD) using horizontal drilling and hydraulic fracturing since the early 2000s has enabled large-scale extraction of hydrocarbons from shale formations, fueling economic growth,^1–4^ but also sparking concerns about freshwater contamination and biodiversity loss.^5–10^ The commercial viability of the USA’s largest unconventional natural gas reservoir, the Marcellus Shale, has driven a prolific increase in shale gas development in the region.^1,11^ Much of this development has taken place within Pennsylvania, where shale gas production has risen from 182 billion cubic feet (BCF) (5.15 billion cubic meters) in 2007 to 7422 BCF (210.17 billion cubic meters) in 2024.^12^

The plausibility of various UOGD contamination pathways and their impacts on groundwater and surface water have been widely examined, with particular emphasis on rivers, as streams are generally considered the first to respond to UOGD-related impacts.^7^ Large-scale statistical analyses in the Marcellus Shale region have found that concentrations of brine-associated chemicals are slightly increased within 1 km of UOGD sites,^8^ while upstream density of UOGD is associated with elevated concentrations of total suspended solids.^9^ Some literature has suggested an association between UOGD and increased stream conductivity,^10,13^ while other studies have observed no relationship between UOGD and any chemical constituents.^14^ Research investigating the biological effects of UOGD showed similarly mixed results. Wastewater discharge associated with UOGD has altered microbial community structure in northwestern Pennsylvania.^15^ Bacterial assemblages in streams near UOGD in northeastern Pennsylvania modeled using co-occurrence networks were less connected and more dominated by pollutant-tolerant taxa.^13^ In contrast, a study in the Pennsylvania State Forest found no significant relationship between UOGD intensity and benthic macroinvertebrate (BMI) communities,^14^ though shifts toward short-lived, generalist taxa have been documented near natural gas activity in Arkansas.^10^ A generalized relationship between UOGD and biological condition has yet to emerge likely due to limited sample sizes, narrow spatial coverage, and confounding land-use stressors such as urbanization and agriculture.

The Marcellus Shale provides a unique opportunity to assess the ecological footprint of energy development at a statewide scale. Both extensive BMI monitoring data and oil and gas development records for Pennsylvania are publicly available, enabling direct evaluation of cumulative impacts across thousands of streams.^16–18^ BMI are particularly valuable for this study because (i) they are a well-established measure of stream health that integrates the cumulative effect of chemical, physical, and biological conditions and respond predictably to stress,^17–19^ (ii) they are known to respond negatively to chemical changes associated with UOGD, such as increased salinity, TSS, and sedimentation,^10,20–23^ and (iii) BMI are collected with standardized sampling protocols and statistically robust subsampling, facilitating large-scale statewide bioassessments.^17,18,24,25^ Despite widespread focus on unconventional shale gas, the long-term ecological footprint of conventional drilling—often denser, older, and less regulated—remains poorly understood.^26^ In this study, we integrate statewide BMI bioassessment data (>6800 samples) with records of UOGD and COGD to estimate the impact of energy development on stream biodiversity. We apply linear mixed models to isolate oil and gas development (OGD) impacts from natural variability associated with site-level characteristics and employ co-occurrence network analysis to assess how energy development reshapes BMI community structure. Our study provides one of the most comprehensive assessments to date of how industrial energy development influences freshwater ecosystems, with implications for understanding cumulative impacts and informing the sustainability of future resource extraction.

## Materials and Methods

### Data

BMI samples taken between 1991 and 2023 from Pennsylvania streams (*n* = 15,107) were downloaded from a publicly accessible database maintained by the Pennsylvania Department of Environmental Protection (PADEP).^16^ All samples were collected with D-framed nets with a 500 μm mesh, subsampled to a target of 200 ± 20, and identified to the genus level if possible, in accordance with PADEP protocols.^24,25^ In wadeable streams, samples were collected along a 100 m length of stream. In semiwadeable streams/rivers, samples were taken along a transect spanning the width of the waterbody to account for heterogeneous mixing of tributaries.^25^ BMI sample locations were snapped to the United States Geological Survey (USGS) stream raster of Pennsylvania,^27^ derived from a 10 m resolution digital elevation model.^27^ Snapped coordinates were input into the USGS StreamStats API^28^ to delineate sample catchments and derive basin characteristics.^29^ The PADEP calculated several taxonomic metrics to describe BMI sample composition, including richness, richness of sensitive Ephemeroptera, Plecoptera, and Trichoptera (EPT) taxa, and Shannon diversity. The PADEP also calculated a composite index of biotic integrity (IBI) score for wadeable and semiwadeable samples, integrating an ensemble of metrics into an overall biological condition score.^17,18^

The dependent variables of interest include taxonomic, functional, and network topology metrics. In particular, taxonomic metrics include richness, richness of sensitive EPT taxa, Shannon diversity, and IBI. Functional metrics include the proportions of each BMI sample belonging to each functional feeding group (FFG). FFG labels and pollution tolerance values were assigned from a supplementary PADEP dataset.^30^ Network topology metrics were calculated using the *igraph* package (v2.2.1)^31,32^ in R (v4.3.0) to quantify the structure of generated co-occurrence networks (more details below). The ecological importance of each metric and its hypothesized relationship with stress, as suggested by the literature, are shown in Table 1.

**Table 1:** Hypothesized Response of Community Metrics to Increased Stress (UOGD and COGD Intensity) Compiled from the Literature^18,19,21,43^

community metric	predicted response	ecological importance
total richness	***–***	total number of unique taxa present in a sample
Shannon diversity	**–**	considers richness and relative abundance (evenness)
mayfly (E) + stonefly (P) + caddisfly (T) taxa richness	**–**	richness of sensitive taxa with pollution tolerance values 0–4
index of biotic integrity (IBI)	***–***	ensemble of various metrics into a single index to represent overall biological condition
% predator	**–**	increased abundance of live prey
% shredder	**–**	increased abundance of coarse particulate organic matter (CPOM) derived from the riparian zone
% filter–collector	**+**	increased abundance of suspended fine particulate organic matter (FPOM)
% collector–gatherer	**+**	increased abundance of benthic FPOM
% scraper	**–**	increased abundance of single cells and nonfilamentous algae colonies
network size	**–**	number of nodes (unique taxa) within the network
connectance	**+**	how often do the same taxa co-occur with each other (realized edges/theoretical maximum)
modularity	**+**	tendency for network to be partitioned into densely connected subcommunities
mean co-occurrence strength	**+**	correlation strength between nodes
mean pollution tolerance	**+**	stress will disproportionately affect taxa with lower pollution tolerances

OGD data were compiled from states intersecting sample catchments (Figure 1b). Data were retrieved from permitting databases and commercial datasets for Pennsylvania,^33^ New York,^34^ Ohio,^35,36^ West Virginia,^35^ and Maryland.^37^ UOGD was distinguished from COGD based on well records and state-specific regulations (Table 2 and Figure 1a). Well density was calculated by spatially joining locations of active wells with sample catchments and by dividing the number of active wells by the catchment area. Geospatial data preprocessing was conducted in Python (v3.9.13). Only wells installed (i.e., spudded) prior to sampling were considered.

**Figure 1:**
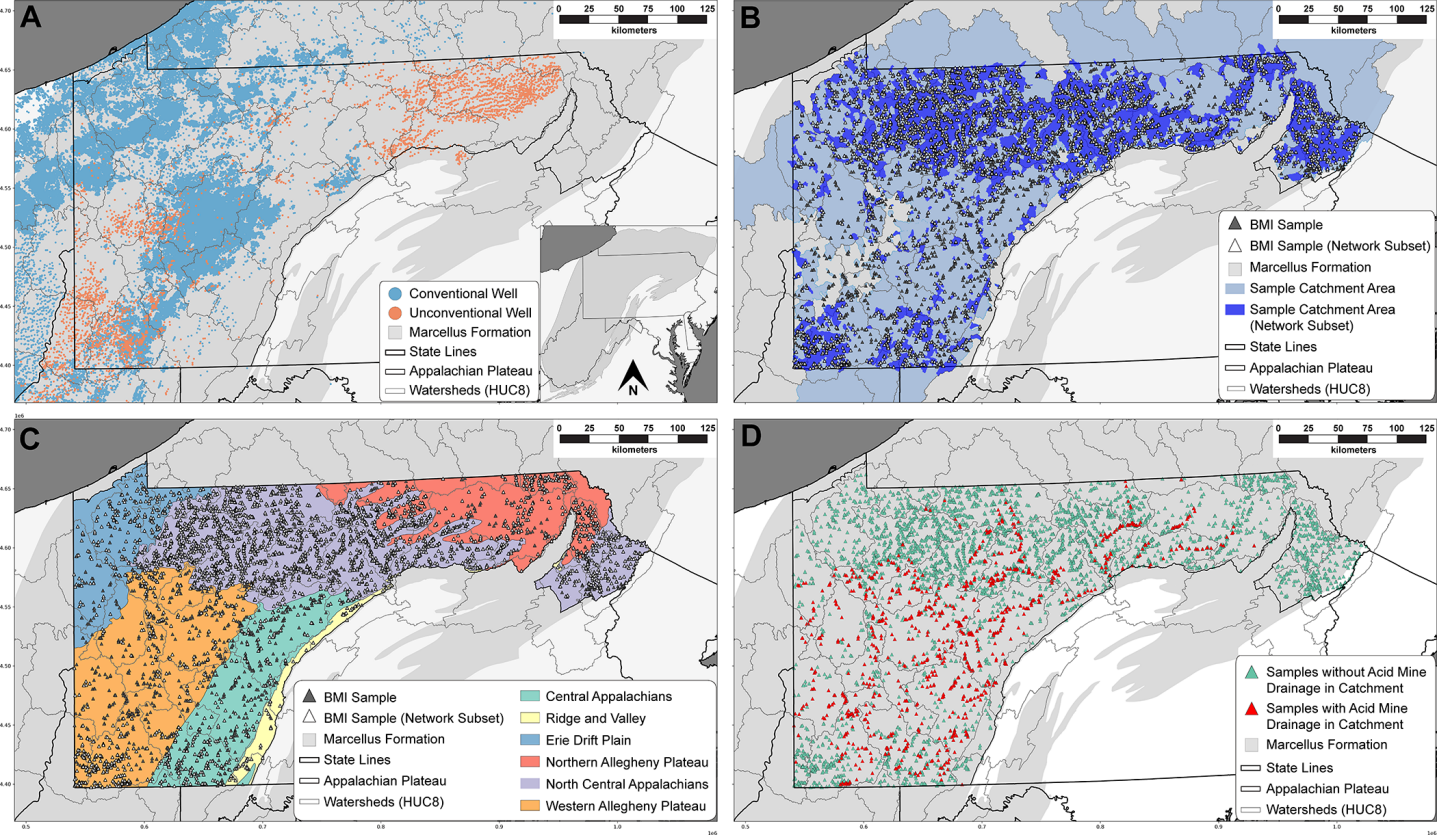
Active conventional and unconventional oil and gas wells within the MSF (A), the spatial extent of all BMI samples and network subset catchments (B), BMI samples labeled by Level III Ecoregion (C), and BMI samples with acid mine drainage present within the catchment area (D).

**Table 2:** Active COG and UOG Wells in States Overlying the Marcellus Shale with Data Sources

state	active well count	source
Pennsylvania	76,952 COG wells	PADEP^33^
	12,841 UOG wells	
New York	16,506 COG wells	NY DEC^34^
West Virginia	51,695 COG wells	Enverus database^35^
	1781 UOG wells	
Ohio	37,934 COG wells	OH DNR^36^/Enverus database^35^
	37 UOG wells	
Maryland	12 COG wells	FracTracker database^37^

### Study Scope and Design

This study focuses on the 6826 BMI samples from wadeable (*n* = 4987) and semiwadeable (*n* = 1839) freestone streams within the Appalachian Plateau (AP) of Pennsylvania (Figure 1). The AP is the physiographic province of interest because it is entirely underpinned by the Marcellus Shale and is the location of nearly all active OGD in Pennsylvania (Figure 1a). Only freestone streams were considered by this study, which are those characterized by the presence of rocky substrate riffles and runs (i.e., areas of high and low turbulence). Freestone streams comprise approximately 95% of streams within the AP and catchment areas range from 0.096 to 9479 square miles (0.249–24,500.50 km^2^). Sampling locations were spatially joined to physiographic^38^ and ecoregion^39^ maps of Pennsylvania to identify samples within the Appalachian Plateau and label each with their respective Level III Ecoregion (Figure 1c). Acid mine drainage (AMD) has also been observed to have a negative effect on biological condition^40^ and was found to be present within the catchment area of a large proportion of BMI samples (*n* = 1701; Figure 1d).^41^ Linear mixed models were built using the *lme4* package^42^ in R to investigate the relationship between variables of interest and OGD presence and intensity.

Models estimating the effect of the OGD intensity on taxonomic and functional metrics were constructed using COGD density, UOGD density, and developed land cover (DLC) as fixed effects and a group of random effects to control for natural variability. This group of random effects, which were used in all models, included ecoregion and AMD presence or absence. The inclusion of these variables as random effects was informed by the significant differences in metric distributions observed between groups (Figures S5 and S6). DLC was considered as a covariate alongside UOGD and COGD to compare the effects of UOGD and COGD to a known stressor of biological condition.^43^ Sampling season was intended to be considered as a random effect to account for significant differences in biological conditions between spring and fall samples. Models failed to converge when season was considered as a random effect due to an insufficient number of samples taken during the fall. As a result, models estimating taxonomic and functional metrics only consider samples taken during the spring (*n* = 5966). Models estimating the effect of OGD presence on the network structure and composition were constructed using COGD presence and UOGD presence as fixed effects, while watershed (hydrologic Unit Code 8; HUC8) was kept as a random effect to account for non-independence and underlying variability in network topology across watersheds (Figure S14).

### Oil and Gas Development on Diversity and Function

High BMI diversity and abundance are indicators of healthy stream conditions, and all taxonomic metrics are expected to decrease with stress (Table 1).^17,18^ BMI taxa are grouped into functional feeding groups (FFG) based on shared morphological and behavioral adaptations for acquiring food. The relative abundance of each FFG has been linked to the availability of their respective food sources.^44^ Generalist groups such as collector–gatherers and filter–collectors tend to tolerate pollution due to their broad diet, whereas specialized groups like shredders and scrapers depend on specific resources that may become depleted under higher stress conditions.^44,45^ Consequently, we predicted that the proportion of collector–gatherers and filter–collectors would increase with stress, while scrapers, shredders, and predators would decline (Table 1). Taxa classified as “unknown” or “piercer” for FFG were excluded due to insufficient representation. COGD density, UOGD density, and % DLC were *z*-score-normalized prior to being used in the models.

### Network Analysis

Because BMI communities have been observed to respond to stream size,^18^ seasonality,^14,18^ AMD presence,^18^ and developed land cover,^43^ we restricted network construction to samples that (i) were collected in spring, (ii) came from wadeable streams, (iii) lacked AMD in the catchment, and (iv) had ≤20% developed land cover. A threshold of 20% DLC was established so that land cover remained relatively consistent across samples, and those heavily impacted by high DLC did not skew the results of network generation (Figure S9). This subset included 3929 samples, with catchment areas ranging from 0.096 to 25.61 sq mi (0.096–66.33 km^2^). Co-occurrence networks were generated using the R package *netassoc* ^46^ following the framework of Simons et al. (2019).^43^ Samples were classified by UOGD and COGD presence/absence, and for each HUC8 with at least 15 samples of a given classification, 10 were randomly subsampled and used to generate a co-occurrence network. Significant co-occurrences were identified by comparing observed networks to null models (100 permutations) of equivalent richness to the observation matrix and retaining edges with a false discovery rate of <10^–4^. This process was repeated 100 times per watershed per group, producing 4500 networks for analysis. Previous studies suggest that networks under greater stress are smaller, have a higher level of connectance, and are dominated by pollution-tolerant taxa.^43^ Metrics to describe the topological structure were calculated for each network. Briefly, network size represents the total number of unique taxa present within the 10 samples, connectance represents the observed number of significant co-occurrences over the theoretical maximum, mean co-occurrence strength reflects the averaged co-occurrence effect size from all edges in the network, and modularity reflects the tendency of a graph to partition into smaller, densely connected subgroups (a more complete explanation of topological metrics and network generation is available in the Supporting Information). Based on the results of other literature employing the same methodology,^43^ we hypothesized that network size would decrease while connectance, mean co-occurrence strength, and modularity would increase in networks where UOGD or COGD was present (Table 1). We further characterize networks by FFG composition and mean pollution tolerance. Mean pollution tolerance was expected to increase with the presence of the OGD, while FFG metrics were expected to respond to OGD presence or absence in the same way they respond to OGD intensity. By controlling for other drivers of the community structure when comparing networks from sites with and without oil and gas development, we aimed to isolate the specific ecological effects of UOGD and COGD. A lack of difference between network structures may suggest that oil and gas development has a limited impact on network structure.

## Results

### Taxonomic and Functional Responses

Taxonomic metrics were scaled using *z*-scores, so that the relative effect of predictors could be compared across our various metrics. At the statewide scale, biological condition was most strongly associated with DLC and COGD, with weaker, nonsignificant effects from UOGD (Figure 2a). Modeling results confirmed that DLC is negatively related to all taxonomic metrics: total richness, Shannon diversity, EPT richness, and IBI (all *p* < 0.001; Figure 2a). COGD intensity was significantly associated with declines in richness (standardized β of −0.05, *p* < 0.001; Figure 2a), EPT richness (standardized β of −0.04, *p* < 0.001; Figure 2a), and IBI (standardized β of −0.03, *p* < 0.01; Figure 2a), although effect sizes were smaller than those of DLC (Figure 2a). By contrast, UOGD intensity was not significantly associated with any taxonomic metrics, although all effects were negative.

**Figure 2:**
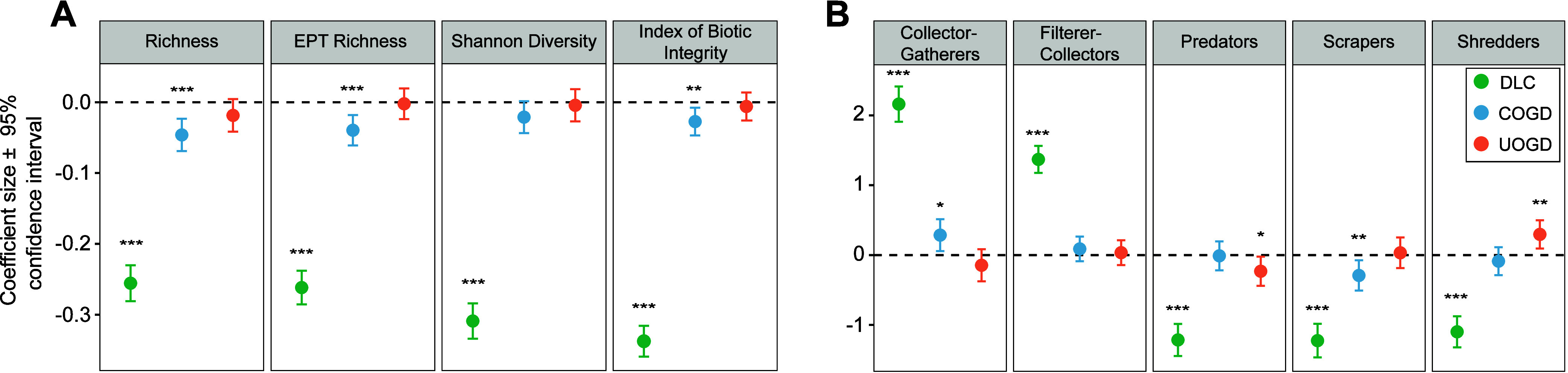
Effect size estimates and 95% confidence intervals for developed land cover (DLC), conventional oil and gas development (COGD), and unconventional oil and gas development (UOGD). Taxonomic metrics (A) were *z*-score-normalized. Functional metrics (B) represent the percentage of samples belonging to each functional feeding group (FFG). The significance of each predictand is represented by asterisks (* = *p* < 0.05, ** = *p* < 0.01, *** = *p* < 0.001).

Functional metrics were also most strongly associated with DLC, followed by COGD and UOGD. In particular, an increase in DLC was significantly associated with increases in the proportion of collector–gatherers (standardized β of 2.16, *p* < 0.001; Figure 2b) and filter–collectors (standardized β of 1.37, *p* < 0.001; Figure 2b) and decreases in the proportions of predators (standardized β of −1.21, *p* < 0.001; Figure 2b), scrapers (standardized β of −1.22, *p* < 0.001; Figure 2b), and shredders (standardized β of −1.10, *p* < 0.001; Figure 2b). Increased COGD density was also associated with increases in the proportion of collector–gatherers (standardized β of 0.29, *p* < 0.05; Figure 2b) and decreases in the proportion of scrapers (standardized β of −0.29, *p* < 0.01; Figure 2b). Increased UOGD density was associated with decreased proportions of predators (standardized β of −0.23, *p* < 0.05; Figure 2b) and increased proportions of shredders (standardized β of 0.30, *p* < 0.01; Figure 2b).

### Network Structure and Functional Feeding Group Composition

Co-occurrence network analyses revealed distinct community structures. Network metrics were normalized with *z*-scores so that the effects of UOGD and COGD could be compared across metrics. From Figure 3a, the presence of COGD was significantly associated with increased network size (standardized β of 0.32, *p* < 0.001; Figure 3a), connectance (standardized β of 0.21, *p* < 0.001; Figure 3a), and mean pollution tolerance (standardized β of 0.40, *p* < 0.001; Figure 3a). UOGD presence was significantly associated with increased network size (standardized β of 0.09, *p* < 0.001; Figure 3a) and mean pollution tolerance (standardized β of 0.22, *p* < 0.001; Figure 3a). UOGD was associated with significantly decreased connectance (standardized β of −0.36, *p* < 0.001; Figure 3a) and mean co-occurrence strength (standardized β of −0.45, *p* < 0.001; Figure 3a). Across all networks, Ordinary Least Squares (OLS) regression using topological network metrics could predict 42% of variation in IBI scores (*p* < 0.001; Figure S11), indicating that changes in the assemblage structure capture a substantial component of an ecological and biological condition.

**Figure 3:**
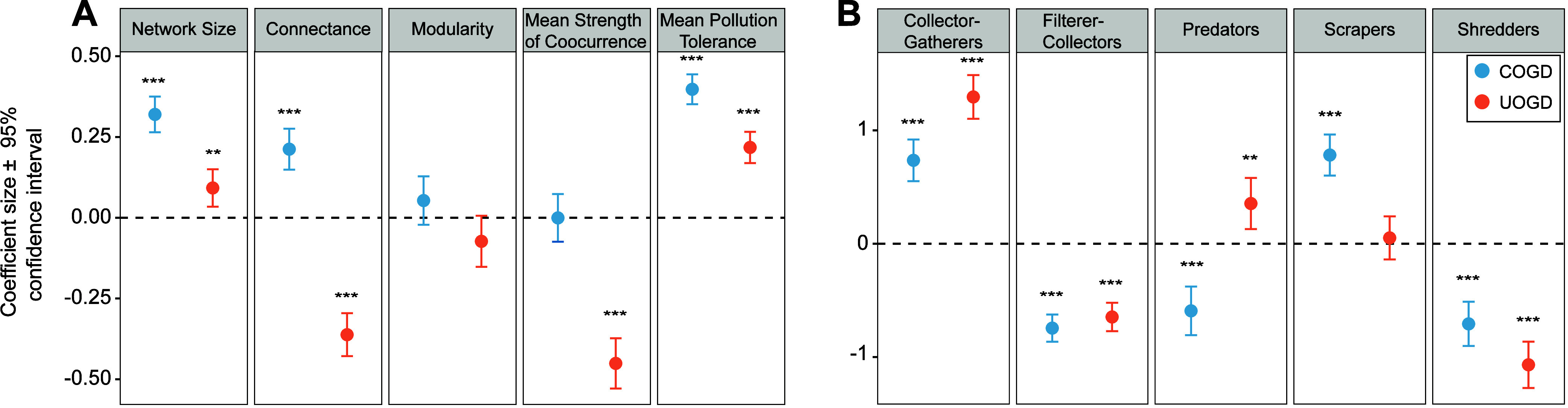
Effect size estimates and 95% confidence intervals for conventional oil and gas development (COGD) and unconventional oil and gas development (UOGD). Network metrics (A) were *z*-score-normalized. Functional metrics (B) represent the percentage of networks belonging to each functional feeding group (FFG). The significance of each predictand is represented by asterisks (* = *p* < 0.05, ** = *p* < 0.01, *** = *p* < 0.001).

Functional composition within networks showed trends similar to those of statewide results with some deviation (Figure 3b). COGD presence was significantly associated with increased proportions of collector–gatherers (standardized β of 0.74, *p* < 0.001; Figure 3b) and scrapers (standardized β of 0.78, *p* < 0.001; Figure 3b) and decreased proportions of filter–collectors (standardized β of −0.75, *p* < 0.001; Figure 3b), predators (standardized β of −0.59, *p* < 0.001; Figure 3b), and shredders (standardized β of −0.71, *p* < 0.001; Figure 3b). UOGD presence was significantly associated with increased proportions of collector–gatherers (standardized β of 1.30, *p* < 0.001; Figure 3b) and predators (standardized β of 0.35, *p* < 0.001; Figure 3b) and decreased proportions of filter–collectors (standardized β of −0.6, *p* < 0.001; Figure 3b) and shredders (standardized β of −1.07, *p* < 0.001: Figure 3b).

## Discussion

### Taxonomic Responses Highlight Disproportionate Impacts of COGD

Together, our results reveal that legacy conventional oil and gas development exerts stronger and broader impacts on stream biodiversity, while unconventional shale gas activity produces weaker but detectable signals. The significant effect of COGD and relative negligible influence of UOGD align with prior analyses from within the Appalachian Basin.^14,26,47^ Additionally, relationships remain significant when models consider only samples from wadeable streams (Figure S13). Several factors may explain why COGD exerts a more pronounced impact. While UOGD has expanded substantially across the Marcellus Shale over the past decade, the average density of UOG wells remains far lower than that of legacy conventional wells.

The strong effects of both DLC and COGD on the richness of pollution-sensitive taxa (Ephemeroptera, Plecoptera, and Trichoptera) underscore their value as ecological indicators and suggest that communities under greater stress experience a replacement of sensitive taxa by more tolerant species capable of persisting in disturbed environments.^18,43^ The similar direction of effects from COGD and DLC likely reflects the shared influence of infrastructure expansion and road use associated with conventional development, whereas the differences in magnitude may result from other land-use pressures, such as urbanization or agriculture, overshadowing the impacts of energy extraction.

Differences between UOGD and COGD effects may stem from differences in the extraction methodology. UOGD and COGD within Pennsylvania have been associated with equivalent amounts of landscape disturbances in previous studies;^47^ however, UOG wells have been shown to produce significantly more energy per unit of disturbed land than COG wells.^26^ In addition, improved OGD policies and regulations with time might lead to more rigorous monitoring of UOG wells compared with older COG wells. Collectively, these findings suggest that despite the disproportionate attention placed on UOGD, remediation and management of legacy conventional drilling sites may be of significantly greater ecological value and would have relatively little impact on energy development.

### Network Analyses Reveal a Divergent Community Structure with UOGD or COGD Presence

Network analysis reveals a more nuanced response in BMI communities to the OGD than suggested by taxonomic results alone. While the negative effect of OGD on richness persists, both UOGD and the COGD are unexpectedly associated with increased network size, contrary to the hypothesized decrease under stress. This pattern, together with a higher mean pollution tolerance in the OGD-affected networks, suggests an enrichment of pollution-tolerant generalists. The two development types diverged in their effects on network connectance: COGD was associated with more connected networks, whereas UOGD was linked to fewer connected networks. Previous literature has attributed increased connectance under stress to a reduction in network size and denser connections among remaining taxa.^43^ Our results, however, show increased connectance alongside larger networks, implying that tolerant taxa co-occur more frequently and evenly in COGD-affected sites. This suggests that COGD may act as an ecological filter for certain sensitive and specialized taxa, contributing to their replacement by pollution-tolerant generalists with broad ecological niches. In contrast, decreased connectance and mean co-occurrence strength under UOGD indicate networks with more taxa that co-occur less often. Taken together, these findings partly contradict initial expectations, suggesting that the presence of UOGD does not negatively affect the BMI assemblage structure. These findings may be associated with the higher distribution of DLC at COGD-affected sites, whereas no difference in DLC was observed between UOGD-present sites and sites with no OGD (Figure S12). This suggests that UOGD may occur in less disturbed, forested areas, where buffering from other stressors (e.g., developed land cover) overshadows UOGD impacts. Similar declines in connectance linked to UOGD have been observed in bacterial networks,^13^ indicating possible fragmentation of the network structure. It is possible that the observed decrease in connectance may be caused by the binary labeling of streams as either an OGD present or absent. High-density UOGD may act as a greater filter on stream composition than the low-density UOGD, decreasing the chances that taxa may co-occur across sites and therefore overall network connectance. Additionally, a linear model composed of the four topological metrics could describe 42% of the variance in IBI scores (Figure S11), lower than reported in previous studies.^43^ The weaker relationship between network structure and biological condition suggests that network structure may decouple from an ecological or biological condition under certain stress regimes or that the signal from binary labeling of OGD presence or absence may be too weak to discern from the effect of stronger confounding variables. Finally, modularity showed no significant changes, consistent with prior research,^43^ reinforcing that network size, connectance, and co-occurrence strength remain the most informative indicators of assemblage robustness.

### OGD Drives the Modest Functional Reorganization in Stream Communities

The functional group responses to the OGD (Figure 2b) highlight how community composition may shift along a gradient of environmental stress. As expected, developed land cover led to increases in generalists (collector–gatherers and filter–collectors) and declines in specialists (predators, scrapers, and shredders). COGD intensity showed similar patterns, with more collector–gatherers and fewer scrapers. UOGD was associated with fewer predators but, unexpectedly, more scrapers. These results indicate some replacement of specialists by generalists may occur under stress, though the small effect sizes suggest that such changes are subtle. Compared with the strong influence of developed land—which consistently emerges as the primary driver of BMI composition—OGD effects are relatively minor.

The PADEP did not consider functional metrics when calculating the IBI due to their limited power in predicting the overall ecological and biological condition.^18^ Nevertheless, these results indicate some reorganization of functional composition associated with the OGD. Estimates based on well presence and intensity were broadly consistent, showing increased collector–gatherers and decreased shredders under both the COGD and UOGD. Interestingly, declines in filter–collectors were also observed, suggesting that OGD may impair the ability to collect particulate matter from the water column. This could be linked to elevated total suspended solids, which previous studies have associated with higher UOGD density in Pennsylvania.^9^ It is important to note that previous studies have also linked COGD to elevated total suspended solids following contamination incidents (e.g., crude oil contamination^48^).

Changes in predator and scraper abundance were inconsistent across models, possibly due to sampling variation or network construction artifacts. Overall, the results suggest that both UOGD and COGD contribute to a modest shift in community composition, characterized by the partial replacement of specialists by generalist collector–gatherers.

### Regional Context

Our findings help reconcile mixed results from prior studies. No relationships were observed between UOGD and taxonomic or functional metrics within the Pennsylvania State Forest, possibly related to stricter drilling management in that region.^14^ In contrast, microbial and bacterial studies elsewhere in Pennsylvania reported significant UOGD effects.^13,15^

These results highlight the need for ensemble approaches to the BMI community analysis. By jointly evaluating taxonomic, functional, and network-level responses, we gained a more comprehensive understanding of how BMI assemblages respond to the OGD than would have been possible using taxonomic metrics alone. With expanded sample sizes and controls for natural variability, we show that UOGD effects—though limited—are detectable at the statewide scale, and that COGD represents a more persistent and widespread stressor (Figures 2 and 3).

These findings underscore that legacy industrial infrastructure can have enduring ecological effects that surpass those of modern, more regulated technologies. By leveraging statewide bioassessment data, this study demonstrates a scalable framework for evaluating cumulative industrial impacts on freshwater ecosystems and determining the sustainability of energy transitions.

## Conclusions

This statewide analysis demonstrates that legacy conventional oil and gas development exerts broader and more persistent ecological impacts on stream biodiversity in comparison to unconventional shale gas development. While unconventional development produced detectable but modest ecological signals, conventional drilling was consistently associated with declines in biodiversity and shifts toward pollution-tolerant taxa. These findings highlight the importance of addressing legacy energy infrastructure in managing freshwater ecosystem health. By integrating large-scale biological monitoring data with development records, this study provides a scalable framework for assessing cumulative ecological impacts and informing sustainable resource management.

## Supplementary Material



## Data Availability

The datasets and codes used in this study are available at 10.5281/zenodo.18497242.

## Notes

Additional materials and methods, network construction details, statistical model descriptions, supplementary figures (Figures S1–S14), and supporting tables (PDF)

## †

California Center for Climate Change Education, West Los Angeles College, Culver City, California 90230, United States
