# Supplementary material for: The Legacy of Conventional Oil and Gas Development Outweighs Shale Gas Impacts on Stream Biodiversity
Source: ACS ES T Water. 2026 Mar 3;6(3):1947–54. doi: 10.1021/acsestwater.5c01413 (PMC12993858; doi:10.1021/acsestwater.5c01413)
Supplement: Supplementary file 1 [file ew5c01413_si_001.pdf]

Supporting Information for

The Legacy of Conventional Oil and Gas Development Outweighs Shale Gas Impacts on Stream Biodiversity

Ryan Olivier-Meehan[1], Ariel Levi Simons[2], Anirudh Prabhu[3], Elizabeth Carter[1,4], Ruta Basijokaite[1], Greg Lackey[5], Tao Wen[1,]*

[1]Department of Earth and Environmental Sciences, Syracuse University, Syracuse, NY 13244, United States

[2]California Center for Climate Change Education, West Los Angeles College, Culver City, CA 90230, United States

[3]Earth and Planets Laboratory, Carnegie Institution for Science, Washington, DC 20015, United States

[4]Department of Civil and Environmental Engineering, Syracuse University, Syracuse, NY 13244, United States

[5]Department of Civil, Environmental and Architectural Engineering, University of Colorado, Boulder, CO 80309, United States

* Corresponding author: Tao Wen (twen08@syr.edu)

This PDF file includes:

Supplementary Text

Supplementary Figures S1 to S14

S1. Detailed Materials and Methods

S1.1 Study Area and Sample Selection

This study investigates benthic macroinvertebrate (BMI) communities from wadeable and semi-wadeable streams within the Appalachian Plateau of Pennsylvania. The Appalachian Plateau is the physiographic province of interest in this study, as it is entirely underlain by the Marcellus Shale Formation and contains 99% of active OGD in Pennsylvania (Figure 1a). This study focuses on the 95% of streams within the Appalachian Plateau that are labeled as freestone, characterized by the presence of rocky substrate riffles and runs (i.e., areas of high and low turbulence)[1,2]. Streams characterized as limestone and multihabitat were not considered in this study, as they were suggested to support and dictate different BMI communities[1,2,3]. While samples considered in the analysis were taken within the Appalachian Plateau, sample catchments frequently extended beyond province and state boundaries. Therefore, the study area was defined as the total spatial extent of all dissolved catchment areas (Figure 1b). OGD data were downloaded from states intersecting with the study area (Figure 1b).

S1.2 Variables of Interest

The variables of interest in this study consist of taxonomic, functional, and network metrics. Taxonomic metrics include: richness – a count of the total number of unique taxa in a sample, Shannon diversity (which measures richness and evenness of taxa within a sample), Ephemeroptera + Plecoptera + Trichoptera (EPT) richness – a count of taxa belonging to these orders whose pollution tolerance value < 4[3,4]. These taxa are generally considered to be sensitive to, or intolerant, of pollution. Index of biotic integrity (IBI) – an ensemble metric derived by the Pennsylvania Department of Environmental Protection (PADEP) that integrates information from various metrics, including those mentioned above, and standardized to control for natural variation due to stream size and sampling season[1,3,4]. Based on previously observed relationships between these taxonomic metrics and stress[3,4], all metrics are expected to decrease with increasing OGD (Table 1).

Functional diversity was represented by the proportions of functional feeding groups (FFG) within each sample. Functional feeding group labels used by the PADEP included: collector-gatherers, filter-collectors, predators, shredders, scrapers, piercers, and unknown. FFG labels were assigned from an associated data dictionary available through the Pennsylvania Spatial Data Access (PASDA)[5]. Taxa identified as either “piercers” or “unknown” were not considered in the statistical evaluation due to a lack of sufficient representation. Previous literature has suggested that proximity to UOGD is associated with increased relative abundances of short-lived generalist taxa and significant shifts in functional feeding group (FFG) composition[6]. It was predicted that proportions of generalist functional groups (collector-gatherers and filter-collectors) would increase while proportions of specialist groups (predators, shredders, scrapers) would decrease with increased OGD intensity (Table 1).

Co-occurrence network structure was quantified through four topological metrics: network size, connectance, mean co-occurrence strength, and modularity. These four metrics describe various aspects of network structure: network size – the number of unique taxa (nodes) present in the samples used to generate the network, connectance- the number of significant co-occurrences between taxa over the theoretical maximum, mean co-occurrence strength- the averaged effect size from all edges within the network, and modularity- the tendency of the network to partition into densely connected subgroups. Network structure under increasing stress has been observed to shift towards smaller, increasingly connected networks that are disproportionately populated by generalist taxa[7]. Additionally, the proportion of nodes belonging to each functional feeding group was calculated for each network alongside the mean pollution tolerance value of all taxa in the network. Pollution tolerance was expected to increase alongside stress.

S1.3 Data Acquisition and Aggregation

Oil and gas data were compiled from Pennsylvania, Ohio, West Virginia, New York, and Maryland to create a unified spatial dataset of active conventional and unconventional wells in states intersecting with the study area. Spud date, coordinate location, well type (conventional or unconventional), and status (active or inactive) were aggregated from a combination of public and commercial databases. Pennsylvania

and New York data were collected from state permitting databases hosted by the PADEP and the New York Department of Environmental Protection. Well data for Ohio were available through the Ohio Department of Natural Resources and merged with data available through the commercial ENVERUS dataset by API number to determine well activity. All West Virginia data were collected from the ENVERUS dataset. Maryland data were downloaded from FracTracker, which previously requested the data from the Maryland government, where they are otherwise available only upon request. All oil and gas activity in New York and Maryland was labeled as conventional, as bans on hydraulic fracturing are active within those states. Well type was not explicitly stated in the dataset for WV, so wells were assumed unconventional if reported as horizontal. Well type was explicitly provided in the data for Ohio and Pennsylvania. Additionally, spud dates were unavailable for Ohio wells, and completion dates were used in place. Active wells with missing spud or completion date were not considered. A breakdown of active conventional and unconventional wells by state, as well as their respective data sources, is shown in Table 2. It is notable that Pennsylvania has more active COG wells and significantly more active UOG wells compared to all other states considered in this study (Table 2).

BMI data were downloaded from the PADEP in two parts. The first dataset contains station metadata (unique station ID, county, stream name, latitude, longitude, hydrologic unit codes (HUC), sampling date, sampling method, and stream type), physical condition scores, and taxonomic diversity metrics for all samples. The second contains counts of unique benthic macroinvertebrates collected from each station. Samples within the Appalachian Plateau were taken between 1991 and 2023, with more than 75% taken after 2009. All samples were collected and subsampled according to PADEP standard protocols. Of the 6,826 samples, all were collected with D-Frame nets with 500-micron mesh with a subsample target 200±20 organisms identified to the lowest taxonomic unit, usually the genus level[1,3,4]. In larger semi-wadeable streams/rivers, a transect protocol was used to account for water quality differences across the width of the profile[4].

Sample catchments were delineated using the US Geological Survey (USGS) StreamStats API to calculate the upstream presence and intensity of COGD and UOGD. Sample coordinates were reprojected

onto the USGS 10 m resolution digital elevation model and manually snapped to the highest accumulation pixel within 10 m and 30 m buffers. The locations of both pixels were preserved and reprojected as latitudinal and longitudinal coordinates and queried to the StreamStats API. Several checks on the API were employed to ensure that the desired catchment data was received. A loop was set with a five-second delay to retry the query five times until a nonzero catchment area was returned. Multithreading and parallel processing were used to increase the query rate, processing 4 queries at a time. The 10 m and 30 m results were compared to the ground truth catchment area provided in the original PADEP dataset. The 30m approach was found to be closer to the true drainage size for the majority (76%) of streams. The closer result was kept if it was within 20% of the original catchment area, a threshold that resulted in a 10% data loss. The loss was likely a function of erroneous snapping amongst the very smallest and largest streams. Catchment areas were returned as polygons alongside basin characteristics such as land cover data. In the case of % developed land, 2001 and 2011 data was provided. Sampling dates were used to pick the most appropriate data for each sample. Locations of active wells were spatially joined to catchment polygons to determine the number of UOG and COG wells within the sample catchment. Presence was determined by > 0 wells within the catchment area, while intensity was calculated by dividing the number of active UOG and COG wells by the catchment area. Only wells spudded prior to the sampling date were considered.

Sampling locations were spatially joined to physiographic[8] and ecoregion[9] to determine samples located within the bounds of the Appalachian Plateau and their respective Level III Ecoregion. Abandoned mine drainage (AMD) sites in PA[10] were spatially joined with sample catchment polygons to determine AMD presence or absence within sample catchments.

S1.4 Biological Condition and Watershed Attributes

Watershed delineation revealed several significant environmental characteristics amongst the samples. Previous literature has suggested that BMI community composition is affected by stream size, sampling season, and acid mine drainage presence[3,4,11]. Non-parametric tests were applied to the full dataset (n = 6,826) to investigate whether the distribution of predictands significantly differed between groups

while accounting for unequal sample sizes and non-normal distributions. Mann-Whitney U tests were used to compare two groups, while Kruskal-Wallis with post-hoc Dunn's tests were used to compare 3 or more groups. 73% of streams within the study area were defined by the PADEP as wadeable (n = 4,987), which generally referred to the ability to wade from one bank to the other[3]. Higher order streams that supported frequent riffle-run habitats were defined as semi-wadeable (n = 1,839)[2,4]. The distribution of upstream drainage area for wadeable streams was significantly smaller than that for semi-wadeable streams (Mann-Whitney $p < 0.001$; Figure S1). Sample catchment areas ranged from 0.096-9,497 sq mi (Figure S2). Wadeable streams had upstream drainage areas < 52 sq mi, while semi-wadeable streams ranged from 25-9,479 sq mi. Catchments of wadeable streams had significantly less developed land cover compared to semi-wadeable streams, likely to represent a higher proportion of headwater streams (Mann-Whitney $p < 0.001$; Figure S1). Distributions of functional and taxonomic metrics, except for richness, in wadeable streams were significantly different than semi-wadeable streams (Mann-Whitney $p < 0.05$; Figure S3). Across all streams, samples taken during the spring (n = 5,966) have significantly higher taxonomic metric values and significantly different functional metric distributions compared to samples taken during the fall (n = 860) (Mann-Whitney $p < 0.001$; Figure S4). Significant differences in taxonomic and functional metrics were also observed depending on the presence or absence of in-catchment AMD (Mann-Whitney $p < 0.001$; Figure S5). Spatial variation was observed in the form of significant differences in taxonomic and functional metrics between samples from different ecoregions (Kruskal-Wallis $p < 0.05$; Figure S6). Histograms showing the distributions of all variables used in modeling are shown in Figure S7. COGD and UOGD density distributions were strongly skewed to the left, reflecting how many catchments have very little UOGD or COGD, while a select few have a significant amount (Figure S7). UOGD density ranged from 0 to 17 wells per sq mi while COGD density was between 0 to 127 wells per sq mi (Figure S7), reflecting the significantly higher abundance of legacy COGD in Pennsylvania, despite the recent boom in UOGD. Significant differences related to sampling season, drainage area, AMD presence, and ecoregion informed their use as fixed effects in LMMs. Preliminary regression analysis was conducted using non-parametric Spearman's rho. Results suggest that DLC, COGD, and UOGD are associated with most

taxonomic and functional metrics, while coefficient sizes suggest that DLC has the strongest effect, followed by COGD, then UOGD (Figure S8, S9). The differences in scale amongst predictors and predictands led to the decision to z-score normalize both prior to modeling.

S1.5 Network Construction

The application of network analysis in community ecology has been increasingly explored, especially as advancements in computing power have made it possible to generate thousands of permutations of co-occurrence networks from relatively simple abundance data[7,12]. Co-occurrence networks are a graphical representation of correlation matrices where nodes denote unique taxa and edges (connections between nodes) denote significant correlations between taxa. Previous literature has found that an ensemble of topological measures describing network structure can explain a significant portion of variation in the California Stream Condition Index, a bioassessment index score similar to the PADEP IBI, and that the network structure undergoes quantifiable changes as a function of increased stress[7]. The subset of data (n = 3,929) used for network generation was (i) from wadeable streams, (ii) had <20% developed land cover, (iii) was collected during the spring (described by the PADEP as November – May[3]), and (iv) had no acid mine drainage within its catchment. Data subsetting was conducted to account for the previously observed effects of attributes such as sampling season, stream size, AMD presence, and DLC (Figures S3-S5, S8). The DLC threshold of 20% was chosen to maintain consistent land cover so that decreased biological condition attributed to high DLC did not skew the results of network analysis (Figure S8). The distributions of watershed attributes and OGD densities for the network subset compared to the full dataset are shown in Figure S2. Notably, distributions of drainage area and DLC are much smaller than in the full dataset (Figure S2). To account for the skewed distributions of UOGD and COGD density, samples were labeled by the presence or absence of UOGD and COGD. Four groups were created by binning our samples this way: those with neither UOGD nor COGD, UOGD only, COGD only, or both. 10 random samples from each group were selected from watersheds with at least 15 samples of a given group and used within the *netassoc* package to generate co-occurrence networks. Sample size (n = 10), threshold (n = 15), and the

reduction of BMI abundance data to presence/absence were conducted based on the methodology of previous literature[7]. With the *make_netassoc_networks* function from the *netassoc* package, the 10 random samples were arranged as a taxa × site matrix (i.e., the observation matrix) to represent all taxa present at the sampled sites. Null matrices were constructed to represent a distribution of taxa co-occurrence expected by chance. 100 null matrices were constructed by preserving the row and column sums while scrambling the values within the original observation matrix. Observation and null matrices were transformed into taxa × taxa matrices to reflect taxa-taxa co-occurrence, and the observed co-occurrence was compared to the null distribution to determine which taxon co-occurs significantly more than would be expected at random. Significant co-occurrences between taxa were preserved if they had a significance level < 10^{-4}. These significant associations were preserved and scaled to the mean and standard deviation of the null distribution. The final matrix contains significant associations between taxa, derived from the 10 randomly sampled samples, and can be represented graphically as a network. Unique taxa are represented graphically as nodes. Edges are drawn between nodes if a significant association exists, while the length of the edge represents the effect size of the association. This process is repeated 100 times per group per HUC8, resulting in 4,500 total networks for statistical analysis. The distributions of network topological metrics (network size, connectance, mean co-occurrence strength, and modularity) were generally observed to be normally distributed, but the scale differed greatly across metrics (Figure S10). To account for this, metrics were normalized prior to modeling so that the relative effects of predictors could be compared across models. The four topological metrics were used as predictors in an ordinary least squares (OLS) linear regression model to predict IBI and determine the relationship between network topology and overall biological condition. All predictors were observed to be significant, and the model accounted for 42% of the variance in IBI ($p < 0.001$; Figure S11). It is worth noting that this is lower than the previously observed relationship between network topology and biological condition, suggesting that the relationship between network structure and stress may not be as strong as in previous studies[7]. Despite the DLC threshold used during

subsetting, it was observed that the distribution of DLC was significantly higher in COGD+ networks compared to all other groups (Kruskal-Wallis $p < 0.001$; Figure S12).

S1.6 Statistical Design

Linear mixed models (LMM) were used to investigate the relationship between variables of interest and OGD presence and intensity. Significant differences observed in metric distribution depending on sampling season, AMD presence/absence, and ecoregion (Figure S3-S6) informed their inclusion as grouping variables in the mixed-effects framework to account for structured variation. Meanwhile UOGD density, COGD density, and the percentage of DLC were included as fixed effects to estimate their overall effects while accounting for group-level variation captured by the random effects. DLC was included as a covariate alongside UOGD and COGD to consider its effect, as well as for context to compare the effect of UOGD and COGD to a known stressor of biological condition[7]. The LMM allows the model the flexibility to use a different intercept for each group defined by the random effects, controlling the natural variation caused by differences between these groups. Sampling season and stream size were originally included as random effects. However, the inclusion of the sampling season caused group sizes to become too small, causing the models to fail to converge. Given the literature review suggests little difference between spring and fall samples[2,3,13,14] and the observation of such relationships within the data (Figure S4), this study only considers spring BMI samples. Stream size was initially included as a fixed effect; however, it did not explain significant additional variance and was removed to simplify the final model. No significant multicollinearity was observed (VIF $\approx$ 1). Relationships between predictors and predictands are maintained even when the models were trained just from wadeable stream samples (Figure S13). LMMs were also used to estimate the effect of OGD presence and absence on network structure. In this instance, HUC8 was kept as a random effect, as significant variation in network topology distribution was observed across different HUC8 watersheds (Figure S14), while UOGD and COGD presence and absence were used as fixed effects.

References

(1) Barbour, M.T.; Gerritsen, J.; Snyder, B.D.; Stribling, J.B. *Rapid Bioassessment Protocols for Use in Streams and Wadeable Rivers: Periphyton, Benthic Macroinvertebrates and Fish, Second Edition*; EPA 841-B-99-002; U.S. Environmental Protection Agency; Office of Water: Washington, D.C, 1999; p 339. https://www.krisweb.com/biblio/gen_usepa_barbouretal_1999_rba.pdf (accessed 2026-02-02).

(2) Pennsylvania Department of Environmental Protection. *Water Quality Monitoring Protocols for Surface Waters*. https://files.dep.state.pa.us/Water/Drinking%20Water%20and%20Facility%20Regulation/WaterQualityPortalFiles/Technical%20Documentation/MONITORING_BOOK.pdf.

(3) Pennsylvania Department of Environmental Protection. *A Benthic Macroinvertebrate Index of Biotic Integrity for Wadeable Freestone Riffle-Run Streams in Pennsylvania*; Pennsylvania Department of Environmental Protection, 2012. https://files.dep.state.pa.us/Water/Drinking%20Water%20and%20Facility%20Regulation/WaterQualityPortalFiles/Technical%20Documentation/freestoneIBImarch2012.pdf (accessed 2024-07-31).

(4) Pennsylvania Department of Environmental Protection. *A BENTHIC MACROINVERTEBRATE MULTIMETRIC INDEX FOR LARGE SEMI-WADEABLE RIVERS: TECHNICAL REPORT*; 2018. https://files.dep.state.pa.us/Water/Drinking%20Water%20and%20Facility%20Regulation/WaterQualityPortalFiles/Technical%20Documentation/LargeRiver_Semiwadeable_Technical_Report.pdf (accessed 2024-08-19).

(5) Pennsylvania Department of Environmental Protection. *PADEP Aquatic Macroinvertebrate Taxa*. https://files.dep.state.pa.us/Water/Drinking%20Water%20and%20Facility%20Regulation/WaterQualityPortalFiles/Methodology/2023%20Methodology/DEP_macroinvertebrate_data_dictionary.html (accessed 2025-03-04).

(6) Johnson, E.; Austin, B. J.; Inlander, E.; Gallipeau, C.; Evans-White, M. A.; Entrekin, S. Stream Macroinvertebrate Communities across a Gradient of Natural Gas Development in the Fayetteville Shale. *Sci. Total Environ.* **2015**, *530–531*, 323–332. https://doi.org/10.1016/j.scitotenv.2015.05.027.

(7) Simons, A. L.; Mazor, R.; Theroux, S. Using Co-occurrence Network Topology in Assessing Ecological Stress in Benthic Macroinvertebrate Communities. *Ecol. Evol.* **2019**, *9* (22), 12789–12801. https://doi.org/10.1002/ece3.5751.

(8) Pennsylvania Department of Conservation and Natural Resources. *Physiographic Provinces*. Pennsylvania Spatial Data Access. https://www.pasda.psu.edu (accessed 2025-03-04).

(9) U.S. Environmental Protection Agency. *Ecoregions of the United States*. https://www.epa.gov/eco-research/ecoregion-download-files-state-region-3 (accessed 2025-03-04).

(10) Pennsylvania Department of Environmental Protection. *AML Inventory Sites 202510*. Pennsylvania Spatial Data Access. https://www.pasda.psu.edu (accessed 2025-03-04).

(11) Cravotta, C. A. Dissolved Metals and Associated Constituents in Abandoned Coal-Mine Discharges, Pennsylvania, USA. Part 1: Constituent Quantities and Correlations. *Appl. Geochem.* **2008**, *23* (2), 166–202. https://doi.org/10.1016/j.apgeochem.2007.10.011.

(12) Morueta-Holme, N.; Blonder, B.; Sandel, B.; McGill, B. J.; Peet, R. K.; Ott, J. E.; Violle, C.; Enquist, B. J.; Jørgensen, P. M.; Svenning, J. A Network Approach for Inferring Species Associations from Co-occurrence Data. *Ecography* **2016**, *39* (12), 1139–1150. https://doi.org/10.1111/ecog.01892.
(13) U.S. Environmental Protection Agency. *What Is the National Rivers and Streams Assessment?*; U.S. Environmental Protection Agency, 2023. https://archive.epa.gov/water/archive/web/html/vms40.html.
(14) Mumford, A. C.; Maloney, K. O.; Akob, D. M.; Nettemann, S.; Proctor, A.; Ditty, J.; Ulsamer, L.; Lookenbill, J.; Cozzarelli, I. M. Shale Gas Development Has Limited Effects on Stream Biology and Geochemistry in a Gradient-Based, Multiparameter Study in Pennsylvania. *Proc. Natl. Acad. Sci.* **2020**, *117* (7), 3670–3677. https://doi.org/10.1073/pnas.1911458117.

SI Figures

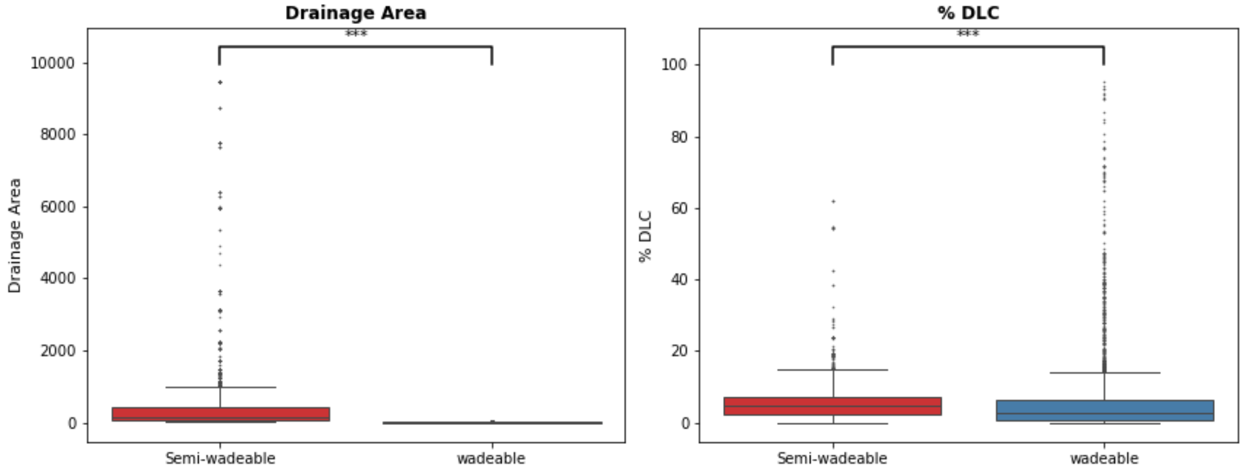

Figure S1. Box-and-whisker plots showing the distribution of drainage size and % developed land cover for wadeable and semi-wadeable sample catchments. Mann-Whitney U tests suggest that semi-wadeable samples have significantly larger drainage areas, of which a greater percentage is covered by developed land. Significant differences are denoted by a line and asterisk (*** indicates $p < 0.001$)

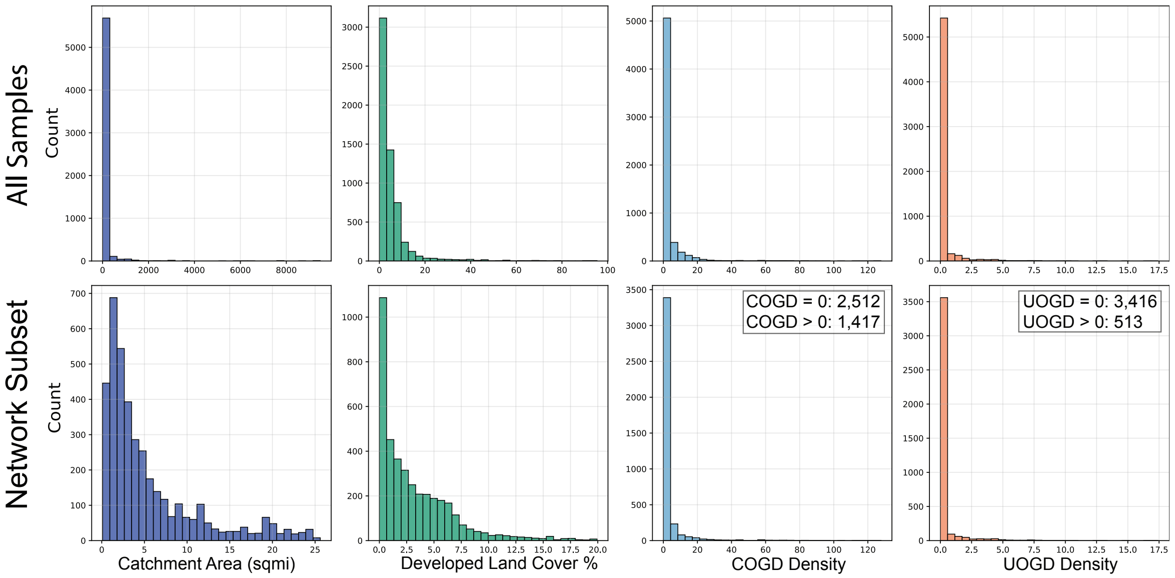

Figure S2. Distribution of catchment area (sq mi), % DLC, COGD density and UOGD density for all samples and samples included in the subset used to generate co-occurrence networks

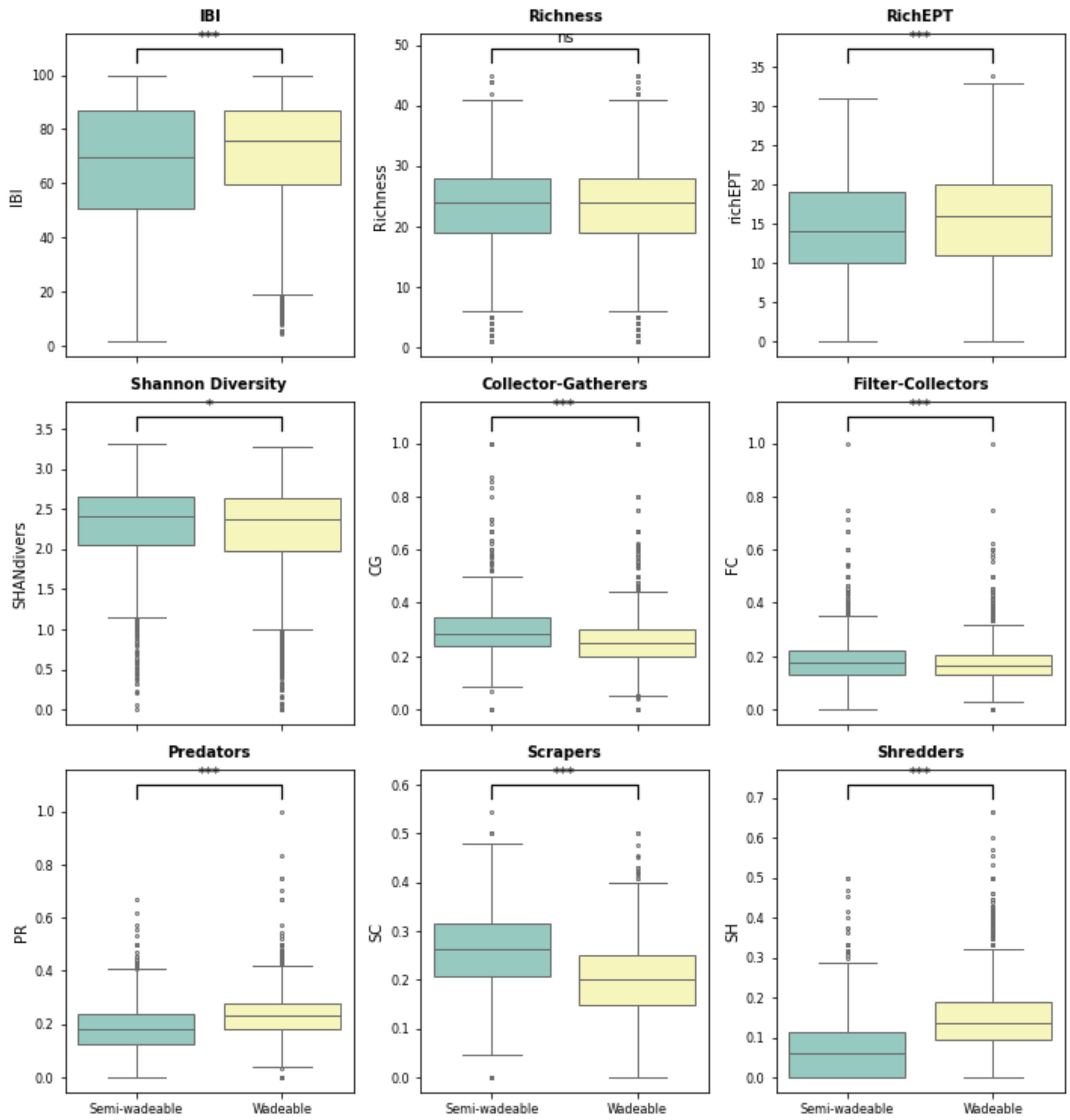

Figure S3. Box-and-whisker plots showing taxonomic and functional diversity metric distributions for wadeable stream samples vs semi-wadeable stream samples. Mann-Whitney U tests suggest significant differences between groups, denoted with asterisks (* = $p < 0.05$, ** = $p < 0.01$, *** = $p < 0.001$)

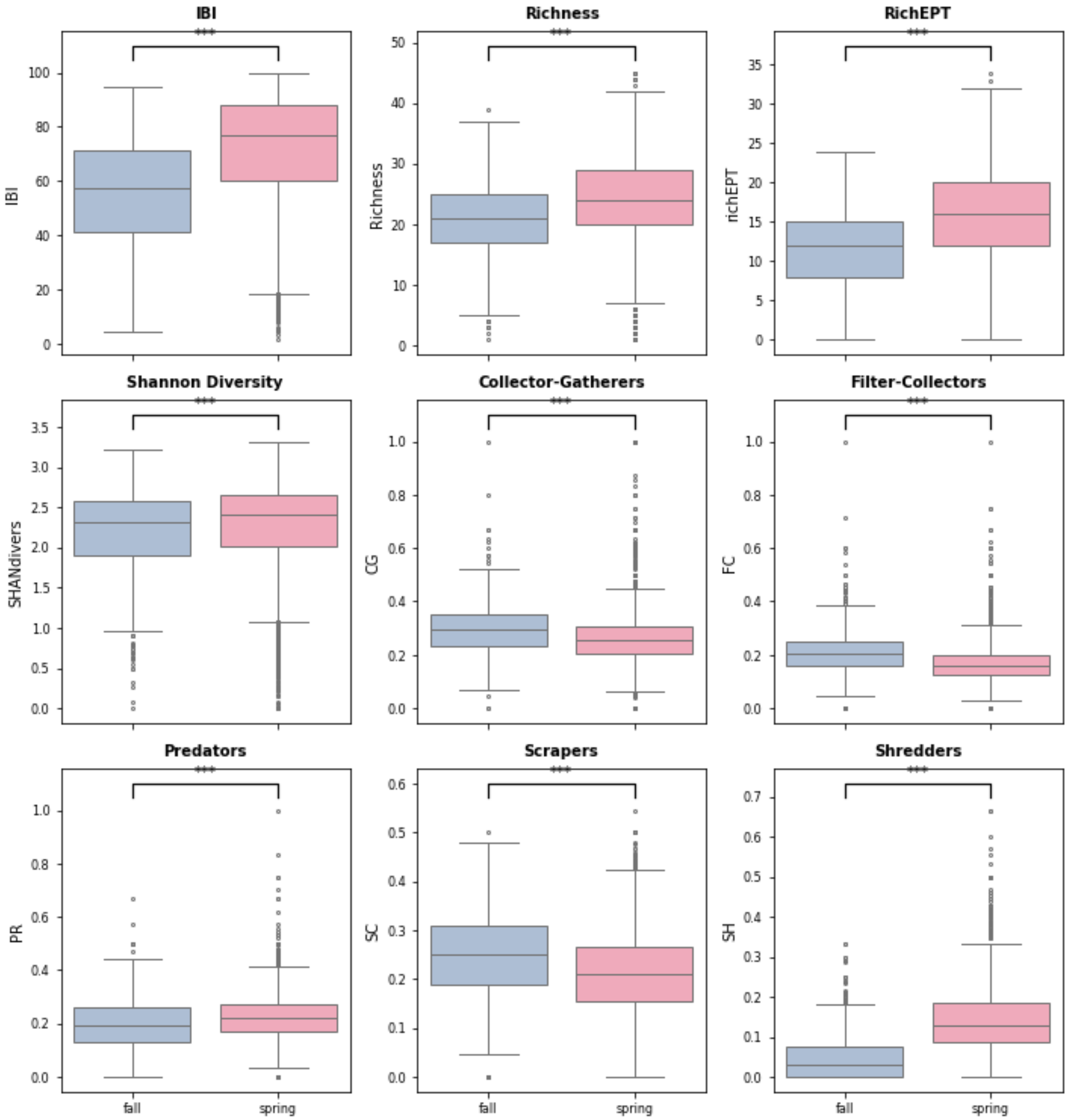

Figure S4. Box-and-whisker plots showing taxonomic and functional diversity metric distributions for fall samples vs spring samples. Mann-Whitney U tests suggest significant differences between groups, denoted with asterisks (* = $p < 0.05$, ** = $p < 0.01$, *** = $p < 0.001$)

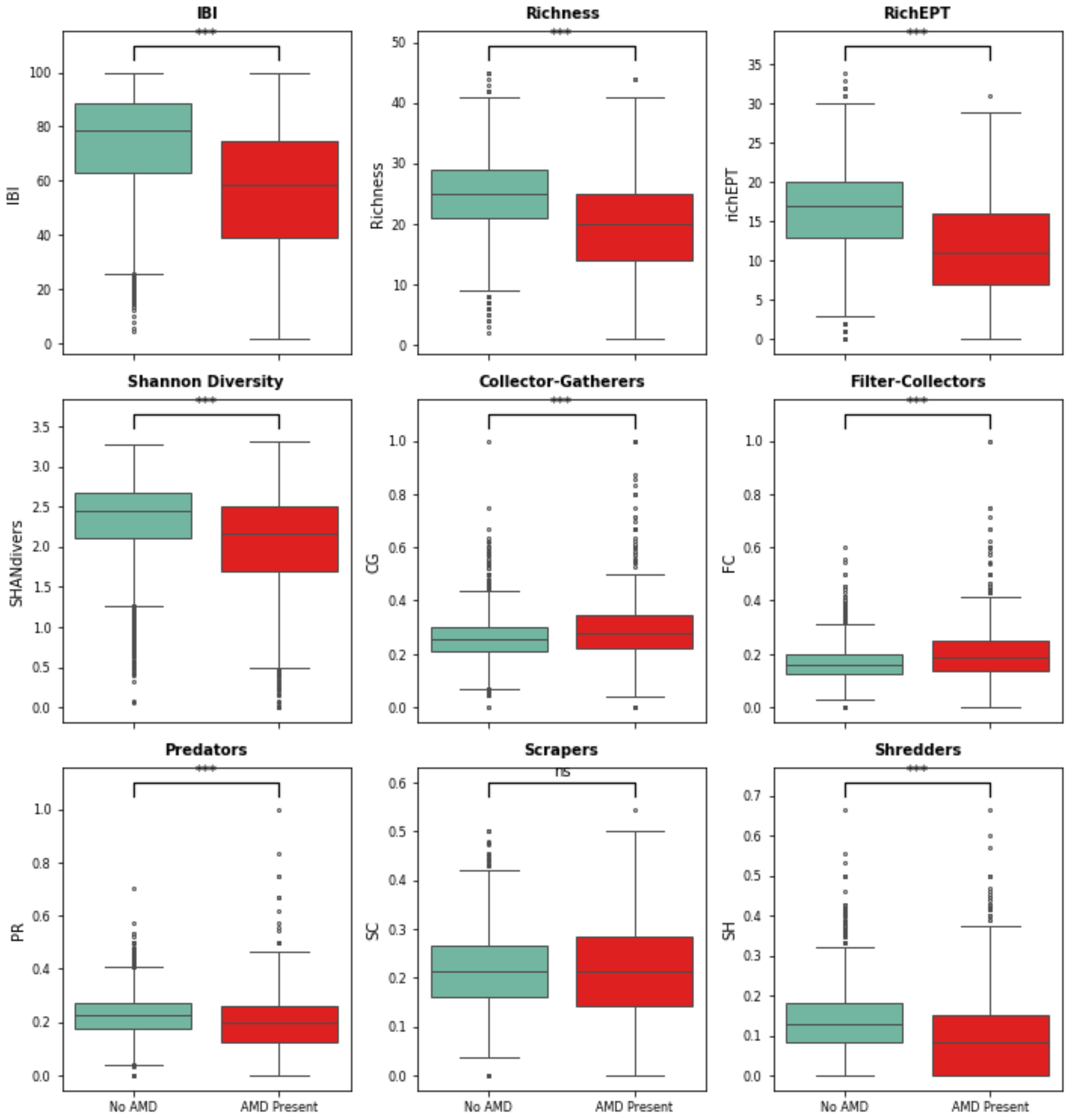

Figure S5. Box-and-whisker plots showing taxonomic and functional diversity metric distributions for samples with AMD present upstream vs samples with no AMD present. Mann-Whitney U tests suggest significant differences between groups, denoted with asterisks (* = $p < 0.05$, ** = $p < 0.01$, *** = $p < 0.001$)

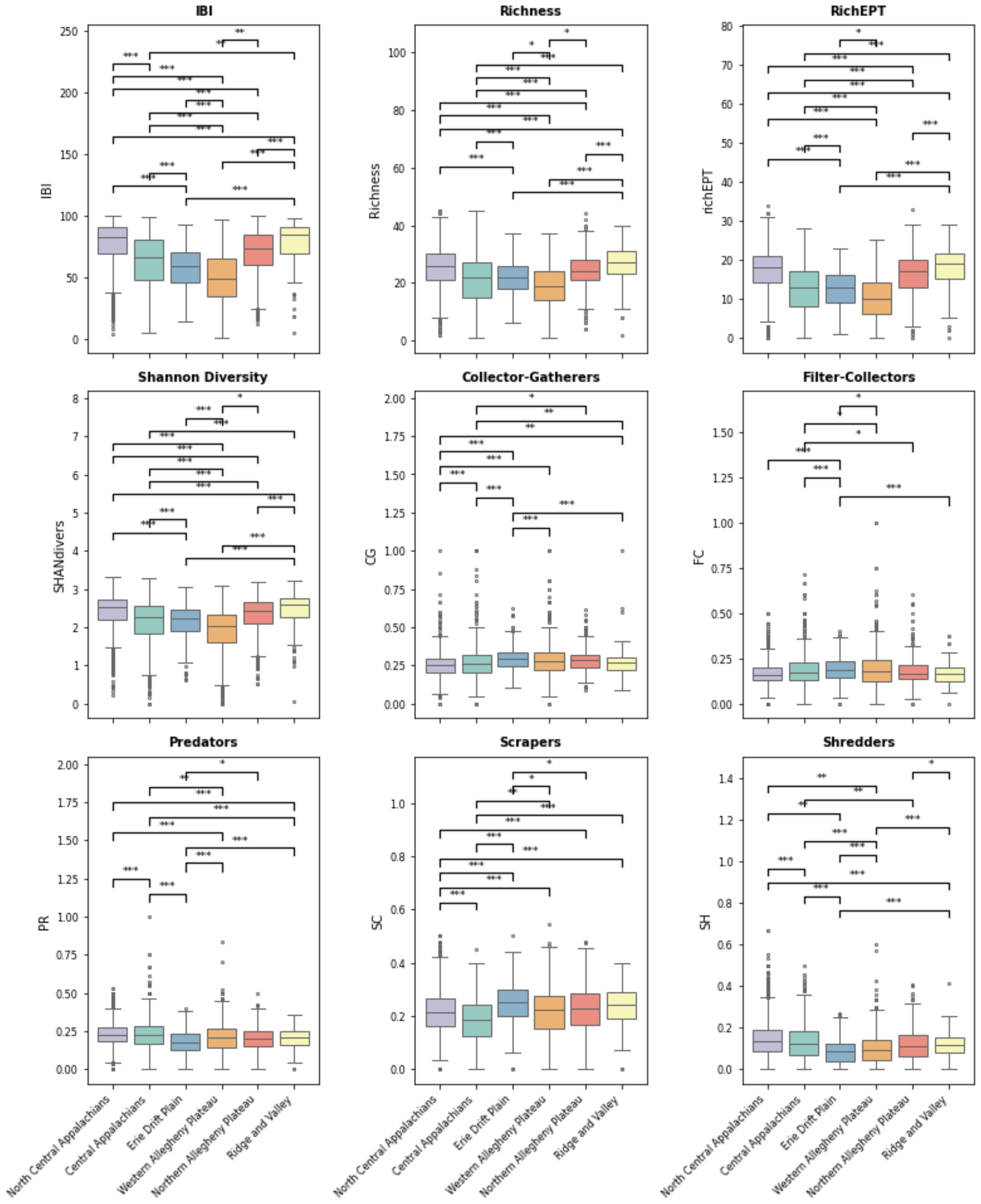

Figure S6. Box-and-whisker plots showing taxonomic and functional diversity metric distributions for samples taken from various ecoregions in Pennsylvania. Kruskal-Wallis and post-hoc Dunn's tests suggest significant differences between groups, denoted with asterisks (* = $p < 0.05$, ** = $p < 0.01$, *** = $p < 0.001$)

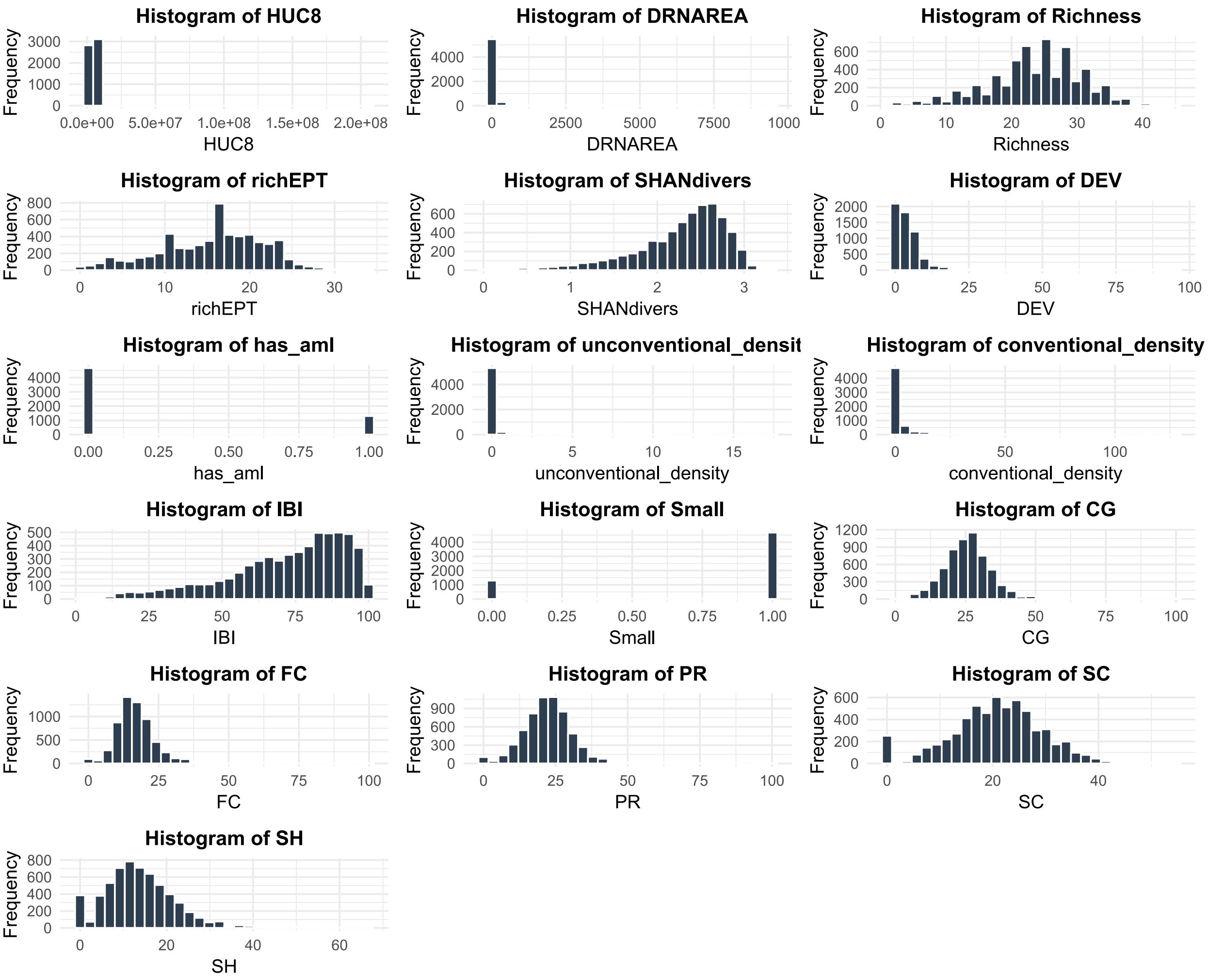

Figure S7. Distributions of predictors and predictand variables whose relationships were estimated with LMMs.

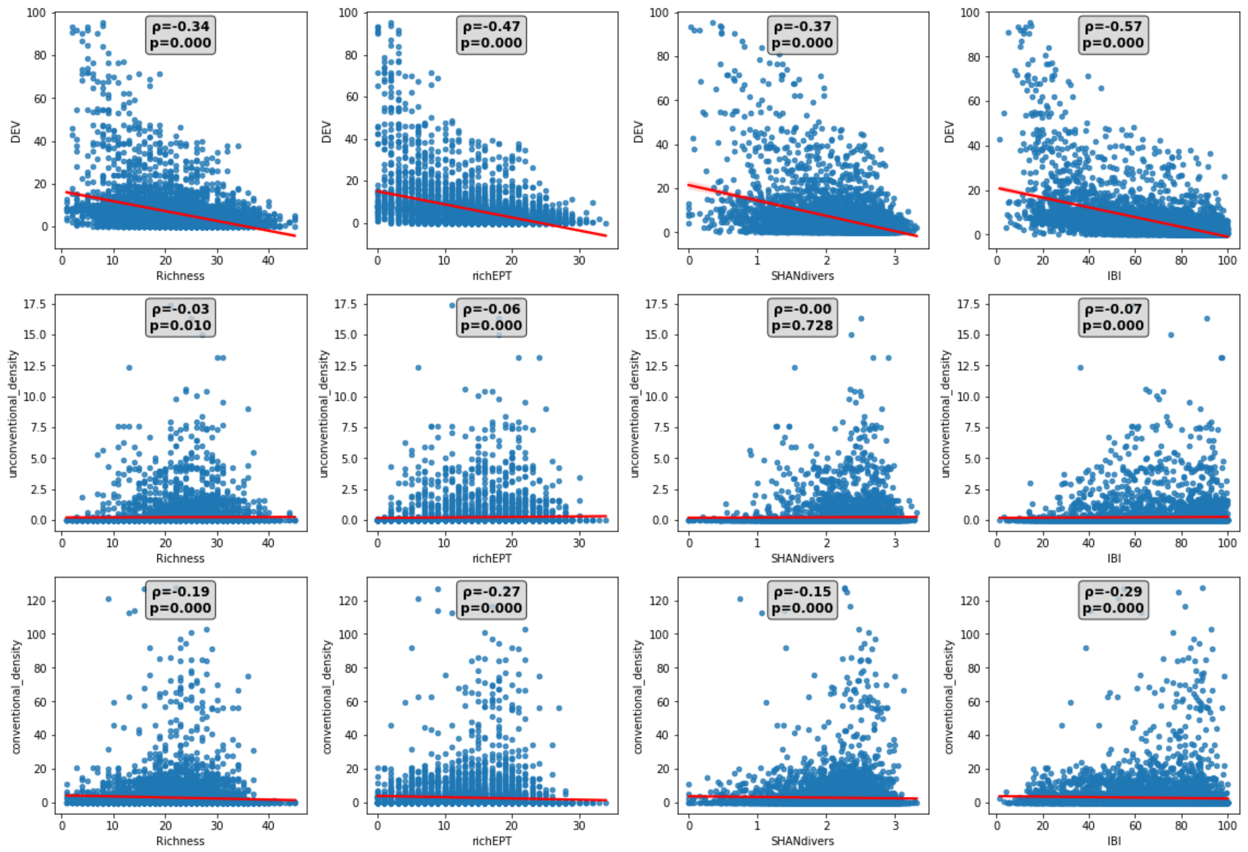

Figure S8. Preliminary regression analysis using Spearman's rho between predictors (% DLC, COGD density, and UOGD density) and taxonomic metrics (richness, richness EPT, Shannon diversity and IBI)

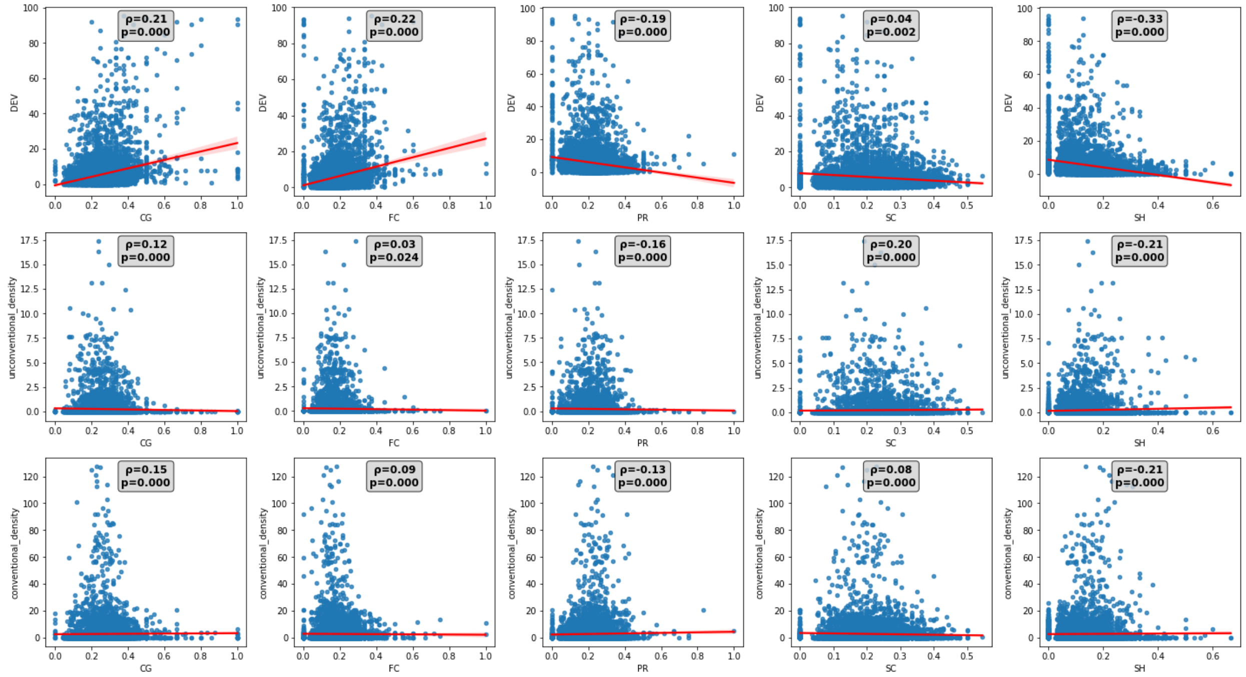

Figure S9. Preliminary regression analysis using Spearman's rho between predictors (% DLC, COGD density, and UOGD density) and functional metrics (collector-gatherers, filter-collectors, predators, scrapers, shredders)

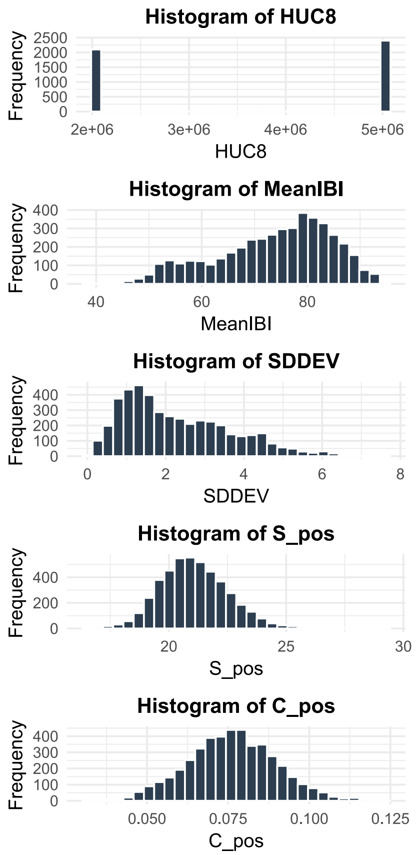

Figure S10. Distributions of network topology metrics and mean watershed attributes calculated during network construction whose relationship with UOGD and COGD presence was estimated with LMMs

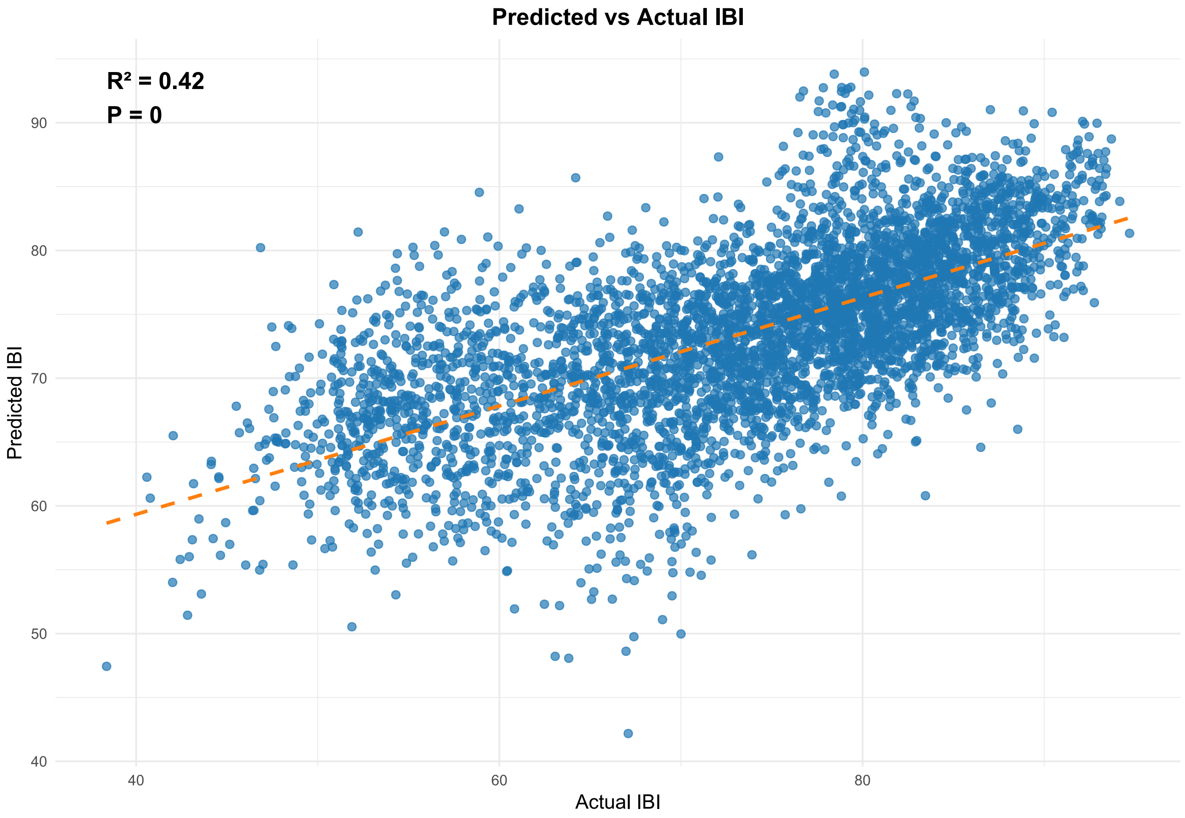

Figure S11. Predicted IBI using OLS regression with network topology metrics over true IBI scores. Pearson's R^2 was used to investigate the relationship between network structure and overall biological condition. Network topology can account for 42% of variation in IBI.

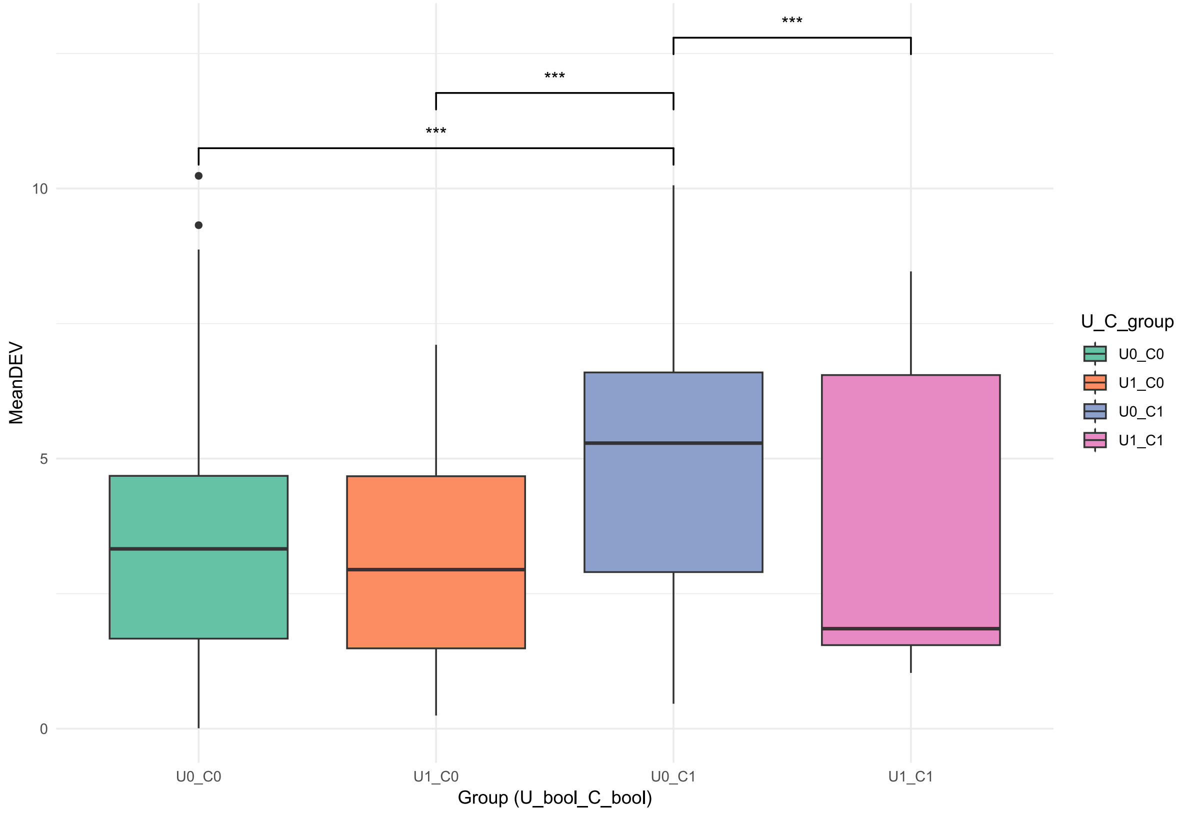

Figure S12. Box-and-whisker plots show the distribution of developed land cover amongst generated networks. Kruskal-Wallis and post-hoc Dunn's tests suggest that COGD+ networks have significantly higher amounts of % DLC, denoted with a line and asterisk (* = $p < 0.05$, ** = $p < 0.01$, *** = $p < 0.001$)

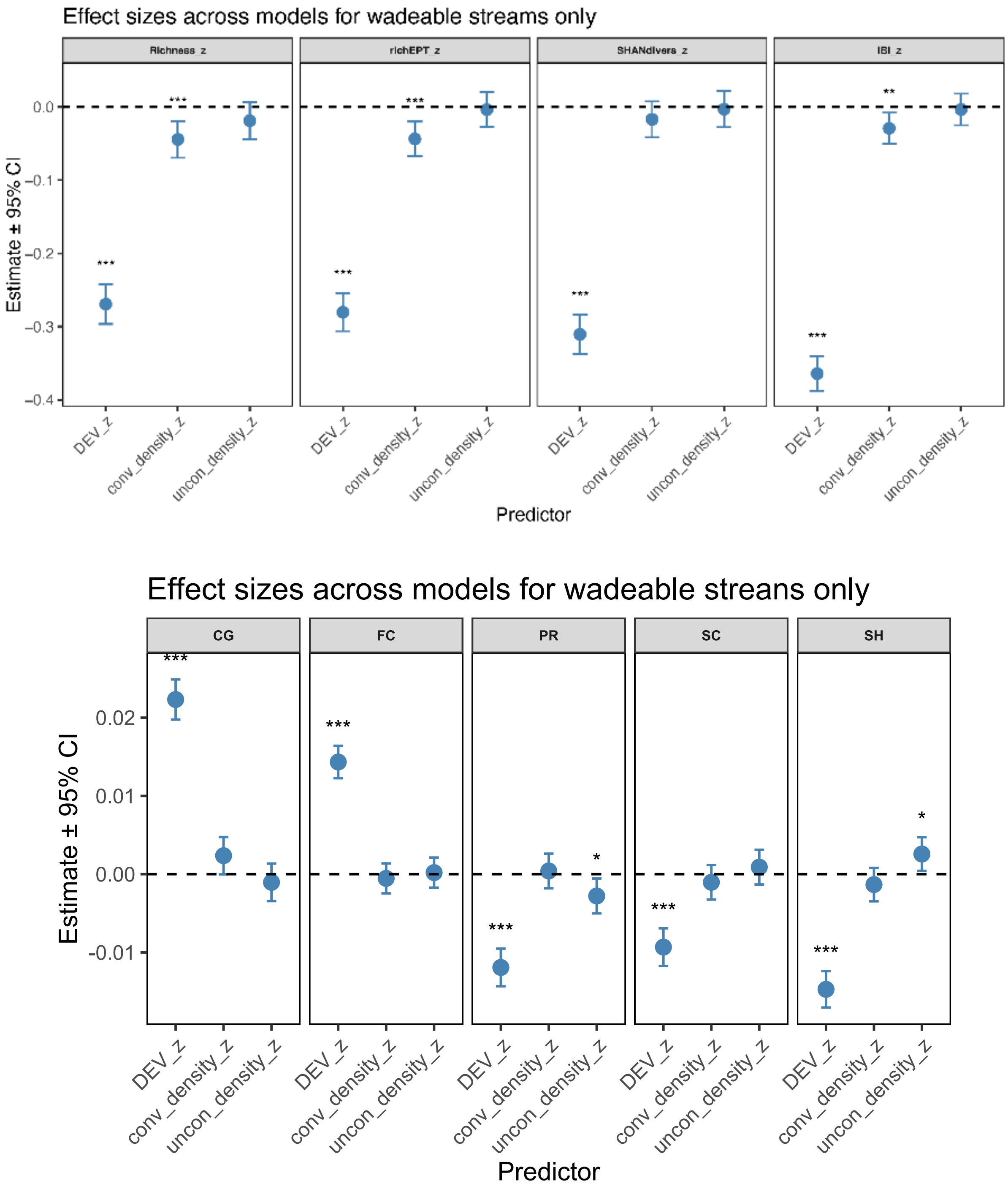

Figure S13. Effect estimates for taxonomic and functional metrics when only wadeable streams are considered. No difference is observed between the results of the model trained on wadeable samples and the model trained on wadeable and semi-wadeable samples.

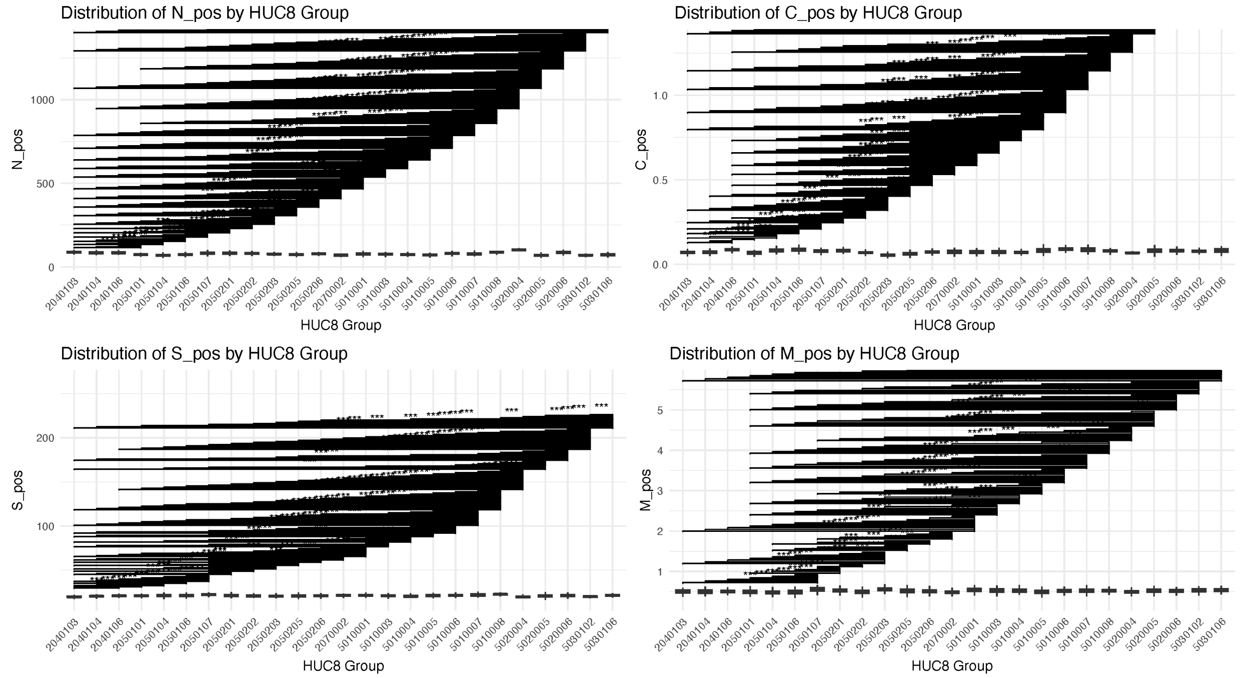

Figure S14. Box-and-whisker plots displaying distributions of network topology metrics and significant differences between HUC8 watersheds determined using Kruskal-Wallis and post-hoc Dunn's tests
